# Effect of methylphenidate on body mass index in children and adolescents in relation to baseline weight status

**DOI:** 10.1007/s00787-026-03070-y

**Published:** 2026-06-27

**Authors:** Julia Izsak, Elin E. Kimland, Jari Martikainen, Elin Dahlén, Linda Halldner, Jenny M. Kindblom

**Affiliations:** 1 Unit of Clinical Pharmacology, Department of Pharmaceuticals, Sahlgrenska University Hospital, Bruna Stråket 21, Gothenburg, Region Västra Götaland 41345 Sweden; 2 Department of Internal Medicine and Clinical Nutrition, Institute of Medicine, The Sahlgrenska Academy, University of Gothenburg, Gothenburg, Sweden; 3 Swedish Medical Products Agency, Uppsala, Sweden; 4 Department of Pharmacy, Faculty of Pharmacy, Uppsala University, Uppsala, Sweden; 5 Bioinformatics and Data Centre, The Sahlgrenska Academy, University of Gothenburg, Gothenburg, Sweden; 6 The NEPI foundation-Network for Pharmacoepidemiology, Stockholm, Sweden; 7 Department of Clinical Sciences, Child and Adolescent Psychiatry, Umeå University, Umeå, Sweden

**Keywords:** Methylphenidate, Body mass index, Weight status, Child

## Abstract

This study aimed to evaluate methylphenidate-associated body mass index (BMI) changes and treatment discontinuation across weight status groups in children and adolescents, using real-world data. We used data from the BMI Epidemiology Study (BEST) Gothenburg cohort linked to the Swedish National Prescribed Drug Register. We included children (< 18 years) initiating methylphenidate, with BMI measurements available at baseline and one year. A subgroup also had BMI measurements at two years. We analysed changes in standardized BMI (zBMI) by baseline weight status and evaluated the impact of dosing and drug discontinuation. Methylphenidate treatment was associated with significant zBMI reduction across all weight status groups in the first treatment year, with the largest decrease observed in those with obesity (-0.5 SD, *p* = 0.01). Higher dose increases, younger age, and female sex were associated with larger zBMI reductions. During the first year, obesity prevalence decreased from 10.4% to 5.7% (*p* < 0.001), while underweight increased from 6.6% to 13.1% (*p* < 0.001). Individuals with underweight or normal weight had higher odds of discontinuation, compared with those with overweight or obesity (OR = 1.73, *p* = 0.004). In the second year, zBMI and weight status remained largely stable. In conclusion, in this real-world setting, methylphenidate was associated with BMI reductions across all weight status groups during the first treatment year. The greatest reduction was seen in individuals with obesity. For those continuing treatment the second year, BMI remained stable.

**Supplementary Information:**

The online version contains supplementary material available at https://doi.org/10.1007/s00787-026-03070-y.

## Introduction

The prevalence of both Attention-Deficit/Hyperactivity Disorder (ADHD) and obesity has increased among children and adolescents over the past decade [1, 2]. There is a significant overlap between ADHD and obesity diagnoses, and growing evidence suggests a complex interplay between the two conditions involving behavioural, neurobiological, and genetic factors [3–5]. Although the underlying mechanisms of this overlap remain incompletely understood, shared dopaminergic dysfunction involving reward processing, has been proposed as one potential common neurobiological pathway between ADHD and obesity [6]. This hypothesis is supported by recent genetic findings indicating shared alterations in dopaminergic signalling between individuals with ADHD and obesity [7].

Methylphenidate, first-line pharmacological treatment for ADHD in children over six years of age, acts through the inhibition of dopamine and norepinephrine reuptake, thereby increasing synaptic concentrations of these neurotransmitters [8, 9]. Given its effect on dopaminergic signalling, it is possible that methylphenidate could exert secondary effects on body weight regulation. Indeed, reduced appetite is a well-documented side effect, and treatment with methylphenidate has been associated with reductions in body mass index (BMI) [10, 11]. However, existing studies examining BMI changes in children and adolescents treated with stimulants have reported somewhat inconsistent findings. Some studies report greater reductions in BMI among children with overweight or obesity [12, 13], including faster decreases among youth with obesity receiving stimulant medication compared to those not treated with stimulants during weight management interventions [14]. In contrast other studies reported more pronounced decreases in children with lower baseline weight [15], or found no significant differences across different weight status groups [16]. Nevertheless, most of these studies have been limited by small sample sizes, selected clinical populations, and insufficient consideration of real-world treatment patterns, such as dosing or discontinuation rates. We have recently shown that in real-world clinical practice, doses of methylphenidate differ depending on a child’s weight status [17], highlighting the need to consider methylphenidate dose as a potential factor influencing BMI outcomes.

We hypothesize that methylphenidate treatment influences body weight across BMI status groups, and that this effect is associated with BMI reductions in individuals with overweight or obesity, and higher discontinuation rates in individuals with underweight. The aim of the present study was to evaluate changes in age- and sex-standardised BMI (zBMI) and discontinuation rates during the first two years of methylphenidate treatment in a population-based cohort of children and adolescents, focusing on differences across weight status groups. We further explore whether changes in zBMI are associated with treatment discontinuation and methylphenidate dosing.

## Methods

In this study, we used data from the BMI Epidemiology Study Gothenburg cohort to evaluate BMI and change in BMI during treatment with methylphenidate. The BMI Epidemiology Study Gothenburg cohort includes objectively measured developmental weight and height data obtained from child and school health care records, as well as information on diagnoses and prescribed medications through linkage to the National Patient Register and the National Prescribed Drug Register [18]. Linkage to registers was performed using the individuals´ Personal Identity Number. The National Prescribed Drug Register, held by the Swedish National Board of Health and Welfare, was initiated in 2005 and includes all prescribed medications that are dispensed at the pharmacy. For simplicity, the term “prescription” is used throughout the manuscript to refer to dispensed prescriptions.

Individuals in the BMI Epidemiology Study Gothenburg cohort were eligible for the present study if they had at least one prescription of methylphenidate dispensed before 18 years of age recorded in the National Prescribed Drug Register before January 31 st, 2021, and in addition had a weight and height measurement available within one year before dispensing. To ensure inclusion of methylphenidate-naïve children and adolescents, we required at least one year free of methylphenidate prescription before inclusion. Methylphenidate was defined using the Anatomical Therapeutic Chemical (ATC) code N06BA04 [19].

### Exposure and background factors

The exposures in this study were methylphenidate treatment and dosing of methylphenidate. We used doses (mg) and weight-adjusted doses (mg/kg) of methylphenidate for the baseline and one-year follow-up prescriptions in children and adolescents with available data, as described earlier [17]. The methylphenidate dose was derived from information on tablet strength and free-text prescription instructions available in the National Prescribed Drug Register and the total daily dose was calculated by summing the tablets prescribed per day. For medication continuation, we used the number of methylphenidate dispensings during the first and second year after the baseline prescription. We defined continuous treatment as three or more dispensings of methylphenidate per year, while treatment discontinuation was defined as two or fewer dispensings per year. This definition was made considering that prescriptions in Sweden are based on yearly prescriptions with a possibility of four dispensings where each dispensing may cover a maximum of three months. Additionally, prescriptions were categorized as extended-release or immediate-release formulations.

To describe psychiatric comorbidity, we used information on psychiatric diagnoses registered in the Swedish National Patient Register, and data on prescribed psychotropic medications from the National Prescribed Drug Register. Psychiatric disorders were defined using the International Classification of Diseases (ICD) system as F00-F99, mood disorders as F30-F39 and intellectual disabilities as F70-79, documented before the baseline methylphenidate prescription. Psychotropic medications were defined using the ATC codes of N05A (antipsychotics), N05B, R06AD (anxiolytics), N06A (antidepressants), and N05C (sedatives), and were considered if dispensed within 6 months before or after the baseline dispensing date of methylphenidate.

### Outcomes

The primary outcome was age- and sex- standardised BMI z-score (zBMI) at one and two years after initiation of methylphenidate treatment. We also assessed shifts between weight status groups by year one and year two after treatment initiation as well as drug discontinuation.

We calculated BMI as weight in kilograms divided by height in meters squared (kg/m^2^) and categorised the values according to age- and sex-specific cutoffs for underweight, overweight and obesity from the International Obesity Task Force for individuals < 18 years of age [20, 21]. According to these cutoffs, underweight was defined as BMI below the corresponding adult BMI < 18.5, overweight as BMI ≥ 25, and obesity as BMI ≥ 30. Children and adolescents not meeting these criteria were classified as normal weight. BMI z-scores were calculated based on the LMS method [20–22].

### Statistical analyses

Descriptive statistics for continuous variables are presented using mean and standard deviation (SD) and categorical variables using number and percentage, if not stated otherwise. We used two-sided paired *t*-tests for continuous variables and McNemar’s tests for categorical variables to compare repeated measurements and proportions within the same individuals between baseline and one year, baseline and two years, and between one and two years, stratified by weight-status groups, sex, and age groups. To assess differences between independent groups, we applied two-sided Welch’s tests for continuous variables and Chi-square tests for categorical variables. Multivariate linear regression models were fitted to evaluate the effect of baseline zBMI, drug dosing, sex, age, drug formulation type and concurrent psychiatric medication on zBMI change from baseline to one year. A logistic regression model was fitted to analyse the association between weight status (underweight/normal weight vs. overweight/obesity) and treatment discontinuation during the first treatment year. Statistical analyses were carried out using SPSS and R. SPSS version 29.0 and R version 4.3.2 (2023-10−31) were used. *P* values < 0.05 were considered statistically significant and are presented as *** *p* < 0.001, ** *p* < 0.01, * *p* < 0.05.

## Results

**Fig. 1:**
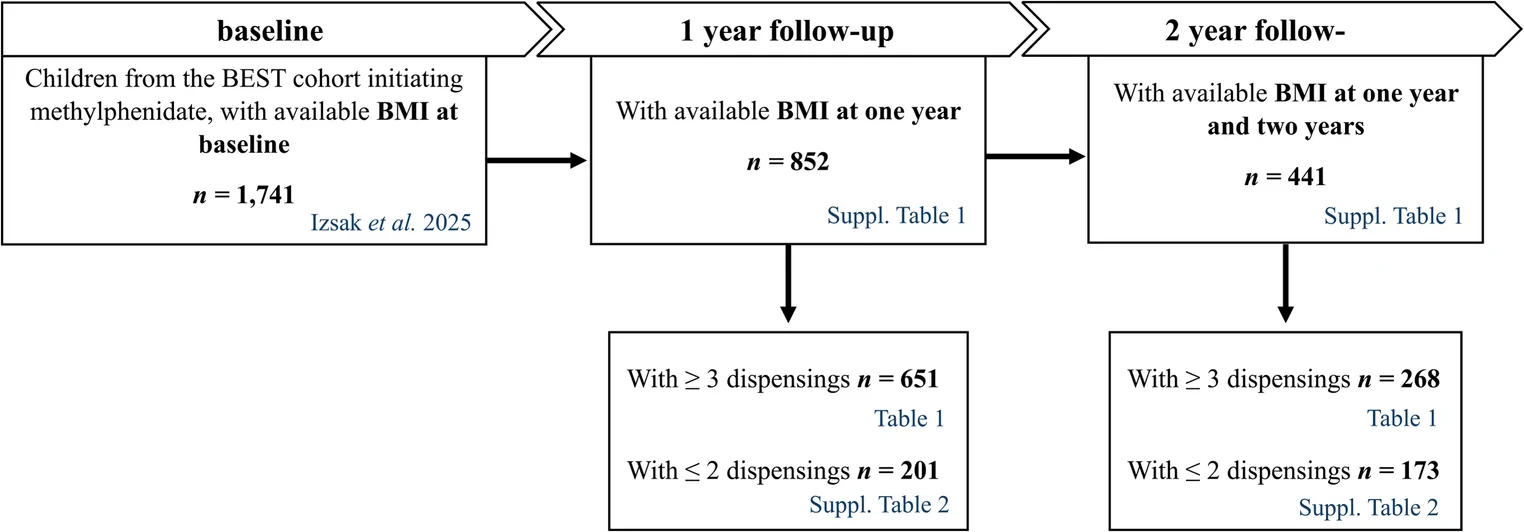
Flow chart of the study cohort. The flow chart summarizes the number of children included across study subgroups. Children with BMI data available at one- and two-year follow-up are detailed in Supplementary Table 1, the subgroup of children with ≥ 3 dispensings during year one or two are detailed in Table 1, and the subgroup of children with 2 or fewer dispensings during year one or two are detailed in Supplementary Table 2. The full cohort of 1,741 children has been described previously [17].

We used the population-based BMI Epidemiology Study Gothenburg cohort to evaluate BMI changes during methylphenidate treatment in relation to baseline weight status in children and adolescents. As previously described, a total of 1,741 children under 18 years of age started methylphenidate treatment during the study period and had baseline height and weight measurements available [17]. The inclusion in the present study is described in the flowchart in Fig. 1. The one-year analysis included children and adolescents with BMI data at baseline and one year (*n* = 852). Among these, 651 children and adolescents had continued treatment, defined as ≥ 3 dispensings within the first year and 201 children and adolescents discontinued (2 or fewer dispensings during the year). The two-year analysis included a subgroup with BMI data available at both one and two years (*n* = 441). Among these, 268 children and adolescents had ≥ 3 dispensings during the second year and 173 children and adolescents had 2 or fewer dispensings. Cohort descriptives at baseline for the individuals with one-year and two-year follow-up data are presented in Table 1 and Supplementary Tables 1 and 2. A subgroup of children and adolescents from the one-year cohort with continuous drug use and complete dosing information available was used to evaluate the association between the daily methylphenidate dose and BMI change. Individuals included in the study had a mean age of approximately 10 years, with a higher proportion of boys and children under 12 years of age (Table 1; Supplementary Table 1; Supplementary Table 2). The majority of the included individuals were prescribed extended-release methylphenidate formulations (*n* = 771, 90.5%) and a smaller proportion received immediate-release formulations (*n* = 81, 9.5%). In the group with one year follow-up, a higher proportion of males compared to females continued treatment (75.9% vs. 68.2%, *p* = 0.03) and there was a lower proportion of underweight (6.6% vs. 13.9%, *p* = 0.002) compared to the group who discontinued. Moreover, a lower proportion had a psychiatric diagnosis prior to the first prescription of methylphenidate (80.2% vs. 86.6%, *p* = 0.05), a lower proportion were treated with antidepressants (4.6% vs. 11.9%, *p* < 0.001) and a lower proportion with anxiolytics (6.0% vs. 13.4%, *p* = 0.001) in the group who continued treatment compared to the group who discontinued.

**Table 1:** Cohort descriptives of children and adolescents initiating methylphenidate who had available BMI and continued drug treatment

Cohort descriptives	Children initiating methylphenidate and had BMI available at baseline and one year and had at least three drug dispensings during the first treatment year (*n* = 651)		Children initiating treatment with methylphenidate who had BMI measurement available at baseline, one year and two years and had at least three drug dispensings during the second treatment year (*n* = 268)	
	Mean (SD)	Range	Mean (SD)	Range
Age at baseline prescription (years)	10.5 (2.8)	4.7–17.9	9.7 (2.5)	4.9–15.5
Height (cm)	143.0 (17.6)	97.0–190.0	138.0 (15.7)	102.0–178.0
Weight (kg)	40.0 (16.3)	14.5–106.0	36.6 (14.9)	16.7–97.3
BMI (kg/m^2^)	18.9 (3.9)	12.6–34.1	18.5 (3.9)	13.0–34.1
BMI z-score (SD)	0.70 (1.17)	−2.69–3.46	0.74 (1.21)	−1.65–3.46
	***n***	**%**	***n***	**%**
Males	494	75.9	200	74.6
Age < 12	455	69.9	220	82.1
Underweight	43	6.6	22	8.2
Overweight without obesity	140	21.5	54	20.1
Obesity	68	10.4	34	12.7
Psychiatric diagnosis before prescription	522	80.2	220	82.1
Mood disorder diagnosis	20	3.1	4	1.5
Intellectual disabilities diagnosis	38	5.8	14	5.2
Treatment with sedatives	133	20.4	52	19.4
Treatment with anxiolytics	39	6.0	15	5.6
Treatment with antidepressants	30	4.6	8	3.0
Treatment with antipsychotics	25	3.8	13	4.9
Baseline prescription	**Mean (SD)**	**Range**	**Mean (SD)**	**Range**
Dose (mg)	15.7 (6.8)	5–54.0	15.5 (7.0)	5–54.0
Dose per kg (mg/kg)	0.43 (0.19)	0.05–1.18	0.46 (0.21)	0.05–1.15

Characteristics of the included children from the BMI Epidemiology Study Gothenburg, below 18 years of age initiating treatment with methylphenidate who also had BMI measurement available at baseline as well as one year after treatment initiation and had at least three drug dispensings during the year (*n* = 651). We further selected children and adolescents who had BMI measurement available at baseline, one and two years after treatment initiation and had at least three drug dispensings during the second treatment year (*n *= 268). Underweight, overweight and obesity were defined according to the international cutoff´s from the International Obesity Task Force [20, 21]. Registered psychiatric diagnoses (F00-F99) were considered before the baseline prescription of methylphenidate. Treatment with sedatives (N05C), anxiolytics (N05B, R06AD), antidepressants (N06A), and antipsychotics (N05A) was considered 6 months before or after the dispensing of methylphenidate. Data are presented as mean (standard deviation [SD]), and range for continuous variables, and as number (%) for categorical variables

*BMI* body mass index, *SD* standard deviation

### Change of zBMI during the first two years of treatment with methylphenidate

**Fig. 2:**
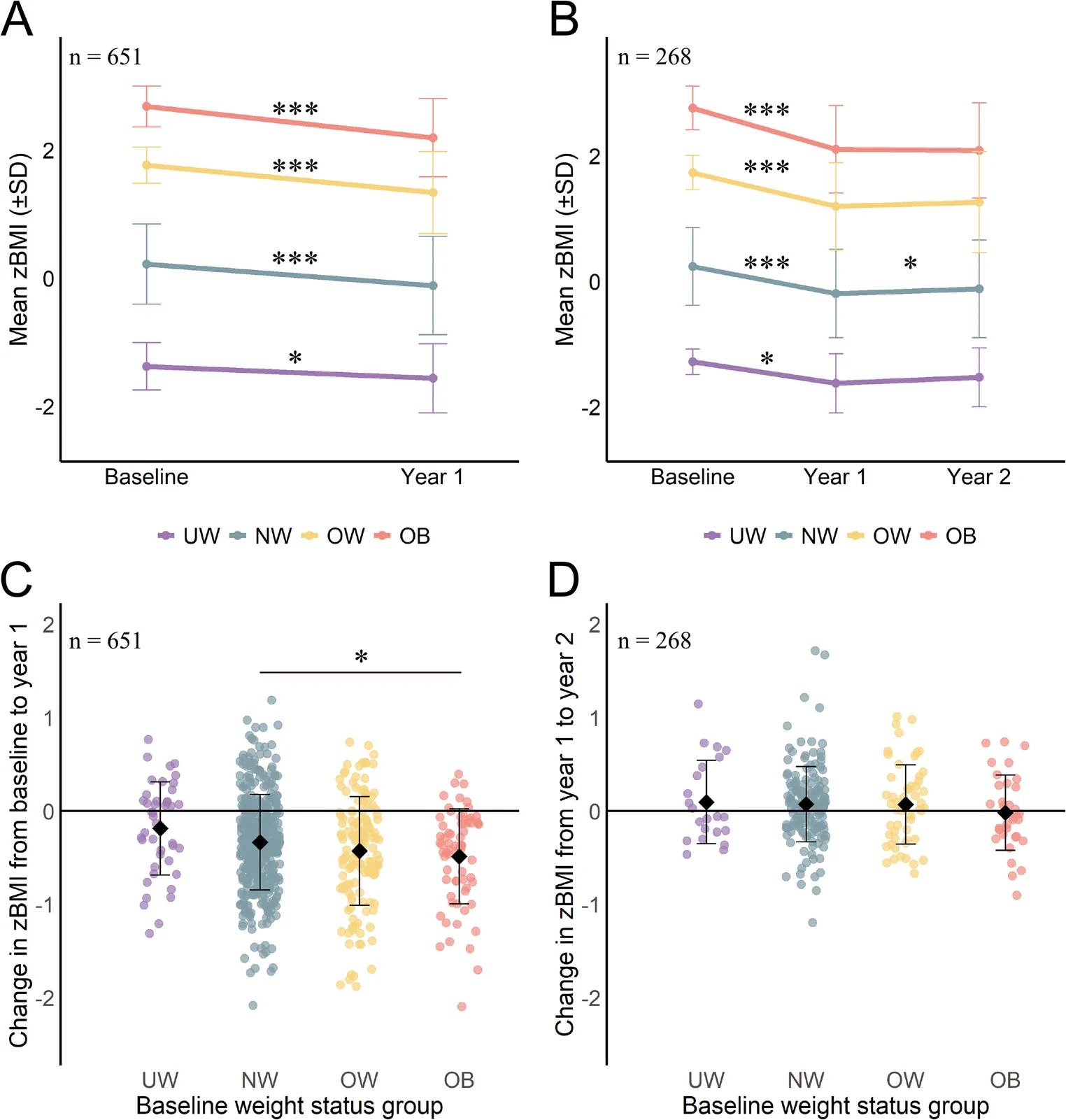
zBMI changes in children and adolescents treated with methylphenidate in relation to weight status during the first two years of treatment. Diagrams show the development of zBMI for the included children from the BMI Epidemiology Study Gothenburg cohort, below 18 years of age initiating treatment with methylphenidate with available BMI measurements and continuing treatment during the first treatment year (*n *= 651) (**A**) and second treatment year (*n *= 268) (**B**). Change in zBMI from baseline to year one (**C**) and year one to year two (**D**) are further visualised in the given subgroups. Underweight, overweight and obesity were defined according to the international cutoff´s from the International Obesity Task Force [20, 21]. Statistical comparisons were performed using two-sided paired *t*-tests, evaluating changes from baseline to one year, and one year to two years within weight status groups in (**A**, **B**) and using two-sided Welch test, comparing the different weight status groups in (**C**, **D**). Statistically significant results are indicated as *** *p *< 0.001, ** p < 0.01, and * p< 0.05. NW – normal weight, OB – obesity, OW – overweight, SD – standard deviation, UW – underweight, zBMI – body mass index z-score

We observed a significant reduction in zBMI during the first treatment year across all baseline weight status groups, as well as across sex and age groups, among children and adolescents who had BMI measurements available and had continuous methylphenidate treatment (*n* = 651) (Table 2; Fig. 2A, B). Children and adolescents who discontinued treatment during the first year (*n* = 201) showed no significant change in zBMI (Table 2, Panel A). The magnitude of zBMI reduction from baseline to year one was greater in children with baseline obesity, compared to their normal weight peers, and showed a reduction of 0.5 SD, *p* = 0.01 (Table 2, Panel A, Fig. 2C).

**Table 2:**
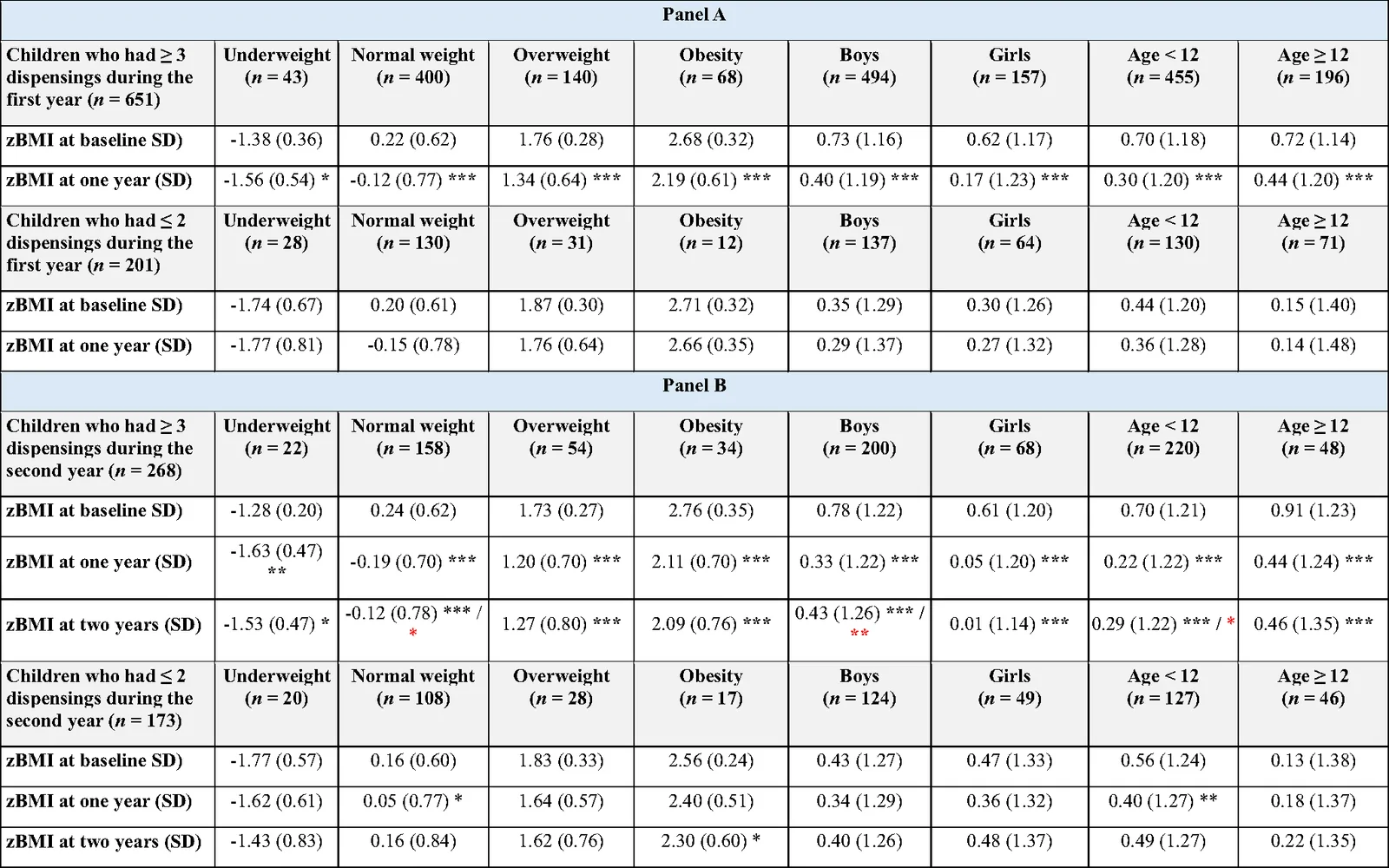
Development of zBMI in children and adolescents initiating methylphenidate in relation to weight status, sex and age during the first two years of treatment

Panel A: Development of zBMI for the included children from the BMI Epidemiology Study Gothenburg cohort, below 18 years of age initiating treatment with methylphenidate who also had BMI measurement available at baseline as well as one year after treatment initiation. Subgroups include children who continued treatment during the first year (≥ 3 dispensings, *n* = 651) and those who discontinued treatment (≤ 2 dispensings, *n* = 201)

Panel B: Development of zBMI for the included children and adolescents who had BMI measurement available at baseline as well as one and two years after treatment initiation. Subgroups include children who continued treatment during the second year (≥ 3 dispensings, *n* = 268) and those who discontinued treatment (≤ 2 dispensings, *n* = 173)

Underweight, overweight and obesity were defined according to the international cutoff´s from the International Obesity Task Force [20, 21]. Statistical comparisons were performed using two-sided paired *t*-tests, evaluating changes from baseline to one year, baseline to two years, and one year to two years within weight status, sex, and age groups. Statistically significant results are indicated as *** *p *< 0.001, ** p < 0.01, and * p < 0.05. Black stars indicate comparisons between baseline and year one or year two, red stars indicate comparisons between year one and year two

*SD* Standard Deviation, *zBMI* body mass index z-score

### Prediction of change in zBMI during methylphenidate treatment

To further investigate prediction of zBMI change from baseline to one year in children and adolescents with continuous drug use (*n* = 595), we fitted two linear regression models with zBMI change (zBMI at one year minus zBMI at baseline) as dependent variable and baseline zBMI, sex, age and either dose change in mg from baseline to follow-up or dose change in mg/kg from baseline to follow-up, as covariates. In the model including dose change per kilogram body weight, inverse associations with zBMI change were observed for baseline zBMI (B = −0.07 zBMI units per SD higher baseline zBMI, 95% confidence interval [CI] −0.11 to −0.04), weight-adjusted dose of methylphenidate (B = −0.48 zBMI units per 1 mg/kg dose increase, 95% CI −0.61 to −0.36) and female sex (B = −0.13 zBMI units for girls vs. boys, 95% CI −0.22 to −0.03). A direct association was seen for age (B = 0.02 zBMI units per 1 year increase in age, 95% CI 0.005 to 0.03). In the model including absolute dose change, results were similar: inverse associations with zBMI change were observed for baseline zBMI (B = −0.06 zBMI units per SD higher baseline zBMI, 95% CI −0.10 to −0.03), dose increases (B = −0.006 zBMI units per 1 mg increase in absolute dose, 95% CI −0.009 to −0.002) and female sex (B = −0.15 zBMI units, 95% CI −0.25 to −0.06). Age (B = 0.03 zBMI units per year increase in age, 95% CI 0.02 to 0.05) displayed a direct association with zBMI change. The model including dose change/kg explained 13.5% of the variance in zBMI change (adjusted R² = 0.135) while the model including absolute dose change explained 6.8% (adjusted R² = 0.068). Adding type of methylphenidate formulation (extended release or immediate release), or concurrent antidepressant, antipsychotic, or anxiolytic medication to the models did not improve the explained variance, and were not significantly associated with zBMI change (data not shown).

During the second treatment year with methylphenidate, the zBMI values remained stable or slightly increased compared to year one (Table 2, Panel B, Fig. 2B, D).

### Shifts in weight status groups during the first two years of treatment with methylphenidate

**Fig. 3:**
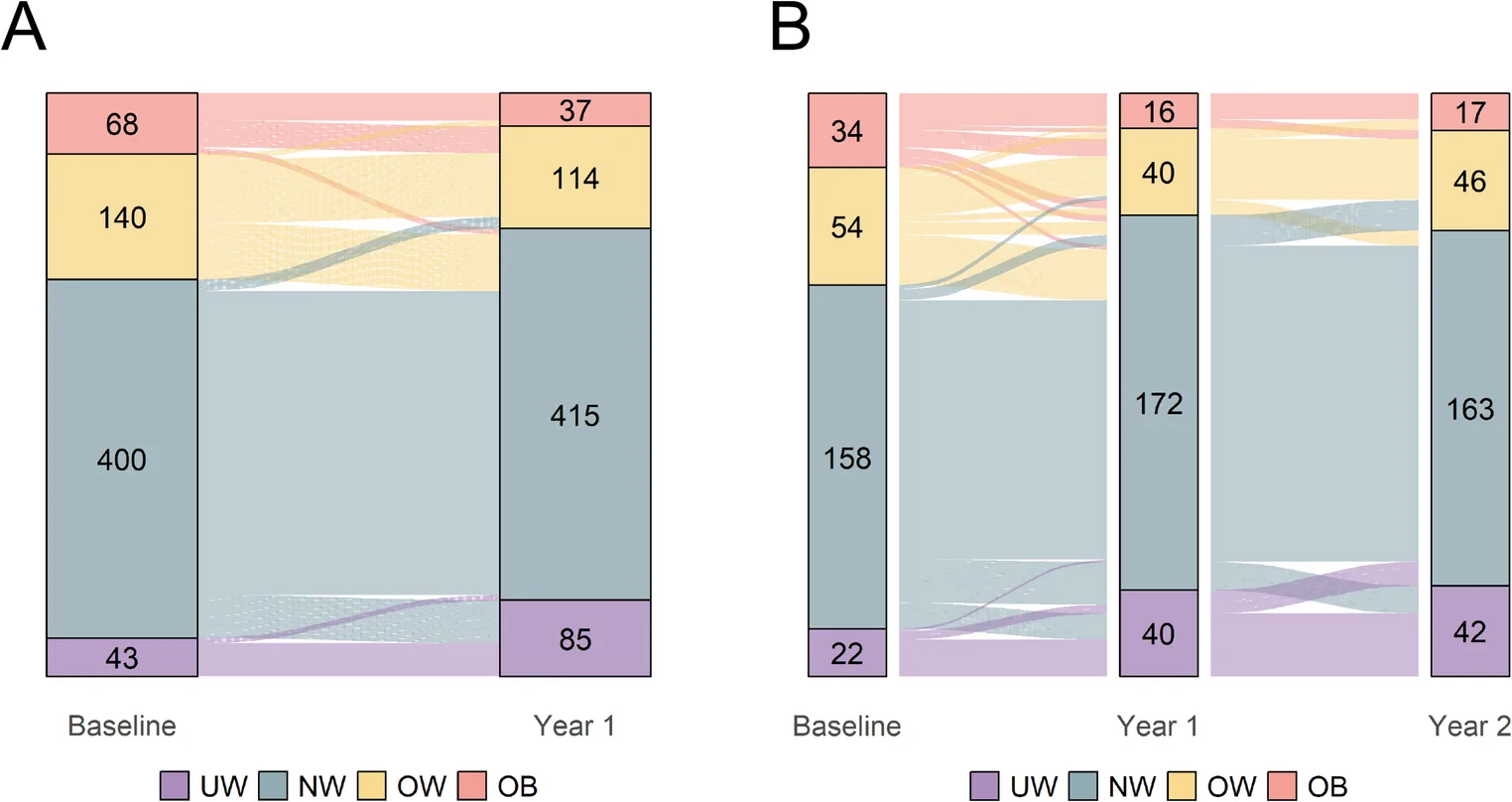
Weight status transitions in children during the first two years after starting methylphenidate. Alluvial diagrams show transitions within weight status groups for the included children (**A**, *n *= 651) from the BMI Epidemiology Study Gothenburg, below 18 years of age initiating treatment with methylphenidate who also had BMI measurement available at baseline as well as one year after treatment initiation. Among the 651 children with available BMI at year one we further selected children (**B**, *n *= 268) who also had BMI measurement available two years after treatment initiation. Underweight, overweight and obesity were defined according to the international cutoff´s from the International Obesity Task Force [20, 21]. The number of patients in the respective weight status group and time point are indicated. NW – normal weight, OB – obesity, OW– overweight, UW – underweight

We next evaluated shifts in weight status among children who initiated methylphenidate, had BMI measurements available one year after treatment start, and had continuous medication (*n* = 651). Among individuals with baseline obesity, 54.4% no children and adolescents from -up (Supplementary Table 3). 44.1% had transitioned to overweight and 10.3% to normal weight. The prevalence of children with obesity decreased from 10.4% at baseline to 5.7% at one year (*p* < 0.001). Among children with baseline overweight, 45.0% shifted to normal weight, contributing to a reduction in overweight prevalence from 21.5% to 17.5% (*p* = 0.02). In contrast, the prevalence of underweight increased from 6.6% at baseline to 13.1% by year one (*p* < 0.001) (Fig. 3, Supplementary Table 3).

During the second year of methylphenidate treatment in children and adolescents with available data (*n* = 268), weight status remained largely stable, with most participants staying in the same category with minor shifts between groups (Fig. 2B, Supplementary Table 3, Panel B).

### Discontinuation

Discontinuation rates during the first treatment year among children and adolescents with available BMI data (*n* = 852) varied by baseline weight status: 15.0% among children with obesity, 18.1% in children with overweight, 24.5% among children with normal weight, and 39.4% in children with underweight (*p* = 0.007 compared to normal weight). In a binary logistic regression model, underweight/normal weight was associated with higher odds of discontinuation compared with the overweight/obesity group (odds ratio [OR] = 1.73, 95% CI 1.19 to 2.51, *p* = 0.004).

### Comparison of individuals who were included versus not included

To assess potential selection bias, baseline characteristics were compared between children included in the one-year follow-up (*n* = 852) and those not included due to missing BMI data (*n* = 889). Children and adolescents included in the one-year follow-up were similar to those not included due to missing BMI data with respect to baseline zBMI, sex, and weight status distribution. However, included individuals were younger (10.6 vs. 11.9 years, *p* < 0.001), had a higher proportion under 12 years of age (68.7% vs. 50.3%, *p* < 0.001), were less frequently diagnosed with mood disorders (3.5% vs. 7.2%, *p* < 0.001), and more frequently diagnosed with intellectual disability (7.9% vs. 5.3%, *p* = 0.03) than those not included.

## Discussion

Given the increasing evidence of overlap between individuals with ADHD and obesity, it is important to understand how methylphenidate, the first-line pharmacological treatment for ADHD, affects BMI in children. In this population-based cohort of children treated with methylphenidate, we found that medication with methylphenidate was associated with a significant decrease in BMI across all weight status groups during the first year of treatment. In children and adolescents continuing treatment, BMI remained stable during the second year. Individuals who discontinued treatment during the first year showed no significant change in zBMI, suggesting that the reduction in BMI observed in the cohort might be related to the methylphenidate treatment. The largest reduction in BMI was observed in the group with obesity. Importantly, over half of the included individuals with obesity at baseline no longer met the criteria for obesity after one year. In parallel, the prevalence of underweight doubled during the first treatment year, warranting careful monitoring of nutritional status among individuals with underweight at start of treatment in future clinical practice. The prevalence of children and adolescents with obesity in our study (9.4–12.7%) was higher than in the background population in Gothenburg, Sweden (2.7–6.1%) [18], which may reflect a higher prevalence of obesity among children and adolescents with ADHD. Indeed, the reported obesity prevalence in children and adolescents with ADHD is ranging from 7% [16, 23], to 13.1% [12] and 16% [13] in different studies, and is consistent with the prevalence seen in our data.

A high baseline zBMI was associated with the largest zBMI reduction. Of note, among individuals with baseline obesity, zBMI declined by 0.5 standard deviations during the first year, an effect size that has been associated with clinically meaningful improvements in cardiometabolic risk factors in children [24]. Our results are in line with some previous studies showing that children with overweight or obesity tend to experience greater BMI reductions from stimulant treatment than those with normal weight [12, 13], and that higher baseline body weight might predict larger reductions [25]. However, not all studies have found similar effects. For example, Dubnov-Raz et al. did not observe a significant BMI change after one year of treatment among children with overweight or obesity [16]. Another observational study found that children with lower baseline z-height and z-weight had greater zBMI reductions over six months [15], while yet another study found no change in zBMI in children with underweight during the first 18 months of treatment [23]. Follow-up times in these studies varied, typically ranging from 6 to 18 months [13, 15, 16, 23, 25], with only one extending to 3 years [12]. None of the studies specifically detailed when the changes in zBMI occurred. Our results indicate that the largest zBMI reductions happened within the first treatment year, with only minor changes thereafter. The data reported by Mellström et al., although not explicitly analysed for timing, also suggest that most zBMI changes occurred during the first 1.5 years of treatment [12]. Clinically, this may be relevant when considering treatment discontinuation due to concerns about further weight loss, as these results suggest that additional BMI reduction after the first treatment year with methylphenidate is minor. Differences in the reported results may further reflect smaller sample sizes or variations in study design, which can limit the ability to detect subgroup effects. The population-based nature of our cohort, including a broader range of clinical profiles and treatment patterns, may partly explain differences with previous studies. Studies using clinical data may include children and adolescents with more severe symptoms or closer medical follow-up, potentially influencing prevalence and treatment effects. Other confounding factors, such as lifestyle or nutritional interventions, may also have contributed to heterogeneity across studies. Moreover, the previous studies usually did not account for discontinuation of treatment during study follow-up. In our cohort, discontinuation rates differed across weight status groups: children with obesity had the lowest rates of discontinuation, and those with underweight had the highest during the first year. This pattern may reflect better tolerance of methylphenidate treatment among children and adolescents with overweight or obesity, potentially due to that the side effect of reduced appetite and weight loss is perceived as favourable. Conversely, higher discontinuation rates among children and adolescents with underweight likely reflect clinical concerns about further weight loss. These concerns related to weight loss in lighter children and adolescents may also partly explain the smaller zBMI reductions observed in this group compared with children with higher baseline zBMI in our study.

Our analysis identified a significant association between increases in absolute doses and weight-adjusted doses of methylphenidate from baseline to follow-up and reductions in zBMI, suggesting that higher doses and doses per kg body weight may be associated with a more pronounced weight effect. This aligns with a previous study that have reported a significant but weak correlation between methylphenidate dose and the degree of BMI reduction in children with ADHD [23]. Dispensing of antidepressants, antipsychotics, or anxiolytics was not associated with BMI outcomes in our cohort, possibly explained by the low number of patients receiving these medications.

In our earlier results, we observed that children with obesity were prescribed slightly higher absolute doses, but lower weight-adjusted doses of methylphenidate compared to their peers with normal weight, whereas children with underweight received lower absolute doses but higher mg/kg doses [17]. Interestingly, despite the lower weight-adjusted doses, children with obesity experienced the most pronounced reductions in zBMI in the present study. This suggests that factors beyond just dose per kg, such as metabolic differences, differences in pharmacodynamic response, or treatment adherence, may contribute to the stronger effect seen in this group. In line with the growing evidence of shared dopaminergic dysfunction between ADHD and obesity [7], the greater BMI response in children with overweight and obesity in our study may also reflect a potential restoration of dopaminergic signalling via methylphenidate’s pharmacological action, potentially contributing to reduced hedonic eating and better appetite regulation. This dopaminergic neurobiological overlap points to a therapeutic target, where children with comorbid ADHD and overweight or obesity may experience dual benefits from methylphenidate treatment. Supporting this notion, a longitudinal study of youth undergoing obesity treatment found that weight loss was slower in those with ADHD compared to those without, but significantly greater among those with ADHD who were treated with stimulant medication compared to those who were not [14]. These findings suggest that improved mesolimbic dopamine signalling may reduce hyperphagia and food-seeking behaviours, although sympathomimetic effects likely also play a role in the observed weight changes in children with obesity treated with methylphenidate. Additionally, methylphenidate reduces maladaptive behaviours associated with ADHD, such as impulsivity and hyperactivity, and improves sustained attention and self-regulation [26]. ADHD symptoms such as impulsivity, hyperactivity and inattention have been described to be correlated with dysregulated eating patterns and increased snacking [27], suggesting that improvements in these domains during treatment with methylphenidate may contribute to more regulated eating behaviours and more consistent meal patterns. Taken together, these potential mechanisms suggest that weight changes during methylphenidate treatment might be multifactorial.

This study has several limitations. The study included children and adolescents treated with methylphenidate and did not include other commonly used stimulant medications. Therefore, the findings of this study cannot be directly generalized to all stimulant medications. We lacked data on ADHD severity, treatment response, side effects, and comorbidities, which may influence treatment continuation and BMI outcomes. Approximately 50% of the original 1741 individuals could not be included in the present study due to unavailability of BMI data at one-year follow-up. We cannot completely exclude selection bias related to treatment-emergent adverse events (such as appetite suppression) that might have led to the discontinuation of both the medication and clinical monitoring. However, baseline BMI and weight status distributions were similar between individuals who were included versus not included, indicating that selection bias based on baseline weight status is likely limited. These factors should nevertheless be considered when interpreting the findings. Further, most patients in this study were treated with extended-release methylphenidate formulations. Although formulation type was not significantly associated with change in zBMI, the relatively small number of patients treated with immediate-release formulations may have limited the power to detect potential differences. Therefore, formulation-specific effects on weight-related outcomes cannot be excluded. Although we applied a one-year wash-out period to include treatment-naïve individuals, we cannot fully rule out prior methylphenidate use. Additionally, we had no information on lifestyle factors such as diet, physical activity, or sleep, which may have an impact on BMI changes. Medication exposure was based on drug dispensings from the pharmacy and may not entirely reflect actual treatment continuation and adherence. This limitation may be especially relevant in a population with ADHD, given dysregulated attention and challenges with prospective memory. As an observational study, the design is subject to inherent bias, and residual confounding cannot be completely ruled out. Finally, our analyses were based on a Swedish cohort, which may limit the generalizability of these findings to other populations.

## Conclusion

In conclusion, methylphenidate was associated with BMI reductions across all weight status groups during the first year of treatment. Children and adolescents with obesity displayed the largest BMI reductions. Further studies are needed to determine whether these effects in children with obesity translate into reduced cardiometabolic risks later in life. Discontinuation rates were highest among individuals with underweight. Careful monitoring may be warranted for individuals with underweight during methylphenidate medication.

## Supplementary Information

Below is the link to the electronic supplementary material.

Supplementary Material 1 (DOCX 53.6 KB)

## Data Availability

The data are not publicly available due to privacy and ethical reasons. Anonymised data can be made available upon reasonable request to the corresponding author and with approval from the University of Gothenburg, in accordance with national law.

## Declarations

### Ehics approval

The study was approved by the ethics committee of the University of Gothenburg, Sweden (D-nr 013-10, on 28 January 2010) with several amendments.
