# Supplementary material for: Effect of methylphenidate on body mass index in children and adolescents in relation to baseline weight status
Source: Eur Child Adolesc Psychiatry. 2026 Jun 27;35(11):3477–86. doi: 10.1007/s00787-026-03070-y (PMC13710619; doi:10.1007/s00787-026-03070-y)
Supplement: Supplementary file 1 [file 787_2026_3070_MOESM1_ESM.docx]

**Effect of methylphenidate on body mass index in children and adolescents in relation to baseline weight status**

Julia Izsak^1,2^, Elin E Kimland^3,4^, Jari Martikainen^5^, Elin Dahlén^4,6^, Linda Halldner^7^, Jenny M Kindblom^1,2^

^1^ Unit of Clinical Pharmacology, Department of Pharmaceuticals, Sahlgrenska University Hospital, Region Västra Götaland, Gothenburg, Sweden

^2^ Department of Internal Medicine and Clinical Nutrition, Institute of Medicine, The Sahlgrenska Academy, University of Gothenburg, Gothenburg, Sweden

^3^ Swedish Medical Products Agency, Uppsala, Sweden

^4^ Department of Pharmacy, Faculty of Pharmacy, Uppsala University, Uppsala, Sweden

^5^ Bioinformatics and Data Centre, The Sahlgrenska Academy, University of Gothenburg, Gothenburg, Sweden

^6^ The NEPI foundation-Network for Pharmacoepidemiology, Stockholm, Sweden

^7^ Department of Clinical Sciences, Child and Adolescent Psychiatry, Umeå University, Umeå, Sweden

**Corresponding author:** Julia Izsak, MD, PhD; Bruna Stråket 21, 41345 Gothenburg, Sweden; +46(0)760838669; [julia.izsak@gu.se](mailto:julia.izsak@gu.se)

**Supplementary Table 1 Cohort descriptives in patients initiating methylphenidate who had available BMI**

| **Cohort descriptives** | **Children initiating methylphenidate with BMI available at baseline and one year (*n* = 852)** | | **Children initiating methylphenidate with BMI available at baseline, one year and two years**  **(*n* = 441)** | |
| --- | --- | --- | --- | --- |
|  | **Mean (SD)** | **Range** | **Mean (SD)** | **Range** |
| Age at baseline prescription (years) | 10.6 (2.9) | 4.5 – 17.9 | 9.9 (2.5) | 4.5 – 16.7 |
| Height (cm) | 142.0 (17.7) | 97.0 – 190.0 | 139.0 (16.3) | 102.0 – 188.0 |
| Weight (kg) | 39.4 (16.0) | 14.5 – 106.0 | 36.5 (14.7) | 16.5 – 101.0 |
| BMI (kg/m^2^) | 18.7 (3.9) | 12.5 – 38.2 | 18.3 (3.8) | 12.5 – 34.1 |
| BMI z-score (SD) | 0.62 (1.20) | -3.80 – 3.46 | 0.62 (1.21) | -2.69 – 3.46 |
|  | ***n*** | **%** | ***n*** | **%** |
| Males | 631 | 74.1 | 324 | 73.5 |
| Age < 12 | 585 | 68.7 | 347 | 78.7 |
| Underweight | 71 | 8.3 | 42 | 9.5 |
| Overweight without obesity | 171 | 20.1 | 82 | 18.6 |
| Obesity | 80 | 9.4 | 51 | 11.6 |
| Psychiatric diagnosis before prescription | 696 | 81.7 | 362 | 82.1 |
| Mood disorder diagnosis | 30 | 3.5 | 9 | 2.0 |
| Intellectual disabilities diagnosis | 67 | 7.9 | 38 | 8.6 |
| Treatment with sedatives | 170 | 20.0 | 88 | 20.0 |
| Treatment with anxiolytics | 66 | 7.7 | 34 | 7.7 |
| Treatment with antidepressants | 54 | 6.3 | 21 | 4.8 |
| Treatment with antipsychotics | 37 | 4.3 | 26 | 5.9 |
| **Baseline prescription** | **Mean (SD)** | **Range** | **Mean (SD)** | **Range** |
| Dose (mg) | 15.5 (6.9) | 2.5 – 54.0 | 15.1 (7.0) | 5 – 54.0 |
| Dose per kg (mg/kg) | 0.43 (0.20) | 0.05 – 1.35 | 0.45 (0.21) | 0.05 – 1.35 |

Baseline characteristics of the included children from the BMI Epidemiology Study Gothenburg cohort, below 18 years of age initiating treatment with methylphenidate, who also had BMI measurement available at baseline as well as one year after treatment initiation (*n* = 852). Among the 852 children with available BMI at year one we further selected children who also had BMI measurement available two years after treatment initiation (*n* = 441). Underweight, overweight and obesity were defined according to the international cutoff´s from the International Obesity Taskforce [1, 2]. Registered psychiatric diagnoses (F00-F99) were considered before the baseline prescription of methylphenidate. Treatment with sedatives (N05C), anxiolytics (N05B, R06AD), antidepressants (N06A), and antipsychotics (N05A) was considered 6 months before or after the dispensing of methylphenidate. Data are presented as mean (standard deviation [SD]), and range for continuous variables, and as number (%) for categorical variables.

BMI – body mass index, SD – standard deviation

**Supplementary Table 2 Cohort descriptives in patients initiating methylphenidate who had available BMI and discontinued drug treatment**

| **Cohort descriptives** | **Children initiating methylphenidate with BMI available at baseline and one year who had ≤ 2 dispensings during the first year (*n* = 201)** | | **Children initiating methylphenidate with BMI available at baseline, one year and two years who had ≤ 2 dispensings during the second year (*n* = 173)** | |
| --- | --- | --- | --- | --- |
|  | **Mean (SD)** | **Range** | **Mean (SD)** | **Range** |
| Age at baseline prescription (years) | 10.7 (3.1) | 4.5 – 17.3 | 10.2 (2.6) | 4.5 – 16.7 |
| Height (cm) | 141.0 (17.9) | 99.5 – 183.0 | 140.0 (17.2) | 103.0 – 188.0 |
| Weight (kg) | 37.6 (15.1) | 16.6 – 104.0 | 36.5 (14.4) | 16.5 – 101.0 |
| BMI (kg/m^2^) | 18.1 (3.8) | 12.5 – 38.2 | 18.1 (3.7) | 12.5 – 32.4 |
| BMI z-score (SD) | 0.34 (1.28) | -3.80 – 3.40 | 0.44 (1.29) | -2.69 – 3.04 |
|  | ***n*** | **%** | ***n*** | **%** |
| Males | 137 | 68.2 | 124 | 71.7 |
| Age < 12 | 130 | 64.7 | 127 | 73.4 |
| Underweight | 28 | 13.9 | 20 | 11.6 |
| Overweight without obesity | 31 | 15.4 | 28 | 16.2 |
| Obesity | 12 | 6.0 | 17 | 9.8 |
| Psychiatric diagnosis before prescription | 174 | 86.6 | 142 | 82.1 |
| Mood disorder diagnosis | 10 | 5 | 5 | 2.9 |
| Intellectual disabilities diagnosis | 29 | 14.4 | 24 | 13.9 |
| Treatment with sedatives | 37 | 18.4 | 36 | 20.8 |
| Treatment with anxiolytics | 27 | 13.4 | 19 | 11.0 |
| Treatment with antidepressants | 24 | 11.9 | 13 | 7.5 |
| Treatment with antipsychotics | 12 | 6.0 | 13 | 7.5 |
| **Baseline prescription** | **Mean (SD)** | **Range** | **Mean (SD)** | **Range** |
| Dose (mg) | 14.9 (7.2) | 2.5 – 54.0 | 14.5 (7.1) | 5 – 54.0 |
| Dose per kg (mg/kg) | 0.43 (0.20) | 0.07 – 1.35 | 0.42 (0.20) | 0.08 – 1.35 |

Baseline characteristics of the included children from the BMI Epidemiology Study Gothenburg cohort, below 18 years of age initiating treatment with methylphenidate, who also had BMI measurement available at baseline as well as one year after treatment initiation and had 2 or fewer dispensings during the first year (*n* = 201). We further selected children who also had BMI measurement available two years after treatment initiation and had 2 or fewer dispensings during the second year (*n* = 173). Underweight, overweight and obesity were defined according to the international cutoff´s from the International Obesity Taskforce [1, 2]. Registered psychiatric diagnoses (F00-F99) were considered before the baseline prescription of methylphenidate. Treatment with sedatives (N05C), anxiolytics (N05B, R06AD), antidepressants (N06A), and antipsychotics (N05A) was considered 6 months before or after the dispensing of methylphenidate. Data are presented as mean (standard deviation [SD]), and range for continuous variables, and as number (%) for categorical variables.

BMI – body mass index, SD – standard deviation

**Supplementary Table 3 Transition matrix of weight status groups in children during the first two years after starting methylphenidate**

| **Panel A** | | | | |
| --- | --- | --- | --- | --- |
| ***n* (%)** | **Year 1** |  |  |  |
| **Baseline** | **Obesity**  (*n* = 37, 5.7%) | **Overweight**  (*n* = 114, 17.5%) | **Normal weight**  (*n* = 415, 63.7%) | **Underweight**  (*n* = 85, 13.1 %) |
| **Obesity**  (*n* = 68, 10.4%) | 31 (45.6) | 30 (44.1) | 7 (10.3) | 0 (0) |
| **Overweight**  (*n* = 140, 21.5%) | 6 (4.3) | 71 (50.7) | 63 (45.0) | 0 (0) |
| **Normal weight**  (*n* = 400, 61.4 %) | 0 (0) | 13 (3.2) | 339 (84.8) | 48 (12.0) |
| **Underweight**  (*n* = 43, 6.6%) | 0 (0) | 0 (0) | 6 (14.0) | 37 (86.0) |
| **Panel B** | | | | |
| ***n* (%)** | **Year 1** |  |  |  |
| **Baseline** | **Obesity**  (*n* = 16, 6.0%) | **Overweight**  (*n* = 40, 14.9%) | **Normal weight**  (*n* = 172, 64.2%) | **Underweight**  (*n* = 40, 14.9%) |
| **Obesity**  (*n* = 34, 12.7%) | 15 (44.1) | 14 (41.2) | 5 (14.7) | 0 (0) |
| **Overweight**  (*n* = 54, 20.1%) | 1 (1.9) | 24 (44.4) | 29 (53.7) | 0 (0) |
| **Normal weight**  (*n* = 158, 59.0%) | 0 (0) | 2 (1.3) | 137 (86.7) | 19 (12.0) |
| **Underweight**  (*n* = 22, 8.2%) | 0 (0) | 0 (0) | 1 (4.5) | 21 (95.5) |
|  | **Year 2** |  |  |  |
| **Year 1** | **Obesity**  (*n* = 17, 6.3%) | **Overweight**  (*n* = 46, 17.2%) | **Normal weight**  (*n* = 163, 60.8%) | **Underweight**  (*n* = 42, 15.7%) |
| **Obesity**  (*n* = 16, 6.0%) | 12 (75.0) | 4 (25.0) | 0 (0) | 0 (0) |
| **Overweight**  (*n* = 40, 14.9%) | 5 (12.5) | 28 (70.0) | 7 (17.5) | 0 (0) |
| **Normal weight**  (*n* = 172, 64.2%) | 0 (0) | 14 (8.1) | 145 (84.3) | 13 (7.6) |
| **Underweight**  (*n* = 40, 14.9%) | 0 (0) | 0 (0) | 11 (27.5) | 29 (72.5) |

Transition matrix of weight status groups in children from the BMI Epidemiology Study Gothenburg cohort initiating treatment with methylphenidate who had BMI measurement available at baseline and one year after treatment start (Panel **A**, *n* = 651) and in children initiating treatment with methylphenidate who had BMI measurement available at baseline, one year and two years after treatment start (Panel **B**, *n* = 268). Numbers and percentages of children in the respective weight status groups are indicated. Underweight, overweight and obesity were defined according to the international cutoff´s from the International Obesity Task Force [1, 2].

**References**

1.	Cole, T.J., et al., *Establishing a standard definition for child overweight and obesity worldwide: international survey.* BMJ, 2000. **320**(7244): p. 1240-3.

2.	Cole, T.J., et al., *Body mass index cut offs to define thinness in children and adolescents: international survey.* BMJ, 2007. **335**(7612): p. 194.
